# Author Correction: Unraveling iron oxides as abiotic catalysts of organic phosphorus recycling in soil and sediment matrices

**DOI:** 10.1038/s41467-024-51063-9

**Published:** 2024-08-02

**Authors:** Jade J. Basinski, Sharon E. Bone, Annaleise R. Klein, Wiriya Thongsomboon, Valerie Mitchell, John T. Shukle, Gregory K. Druschel, Aaron Thompson, Ludmilla Aristilde

**Affiliations:** 1 Department of Civil and Environmental Engineering, Northwestern University, Evanston, IL USA; 2 Stanford Synchrotron Radiation Light Source, SLAC National Accelerator Laboratory, Menlo Park, CA USA; 3 Australian Synchrotron, Australian Nuclear Science and Technology Organisation, Clayton, VIC Australia; 4 Department of Earth Sciences, Indiana University-Purdue University Indianapolis, Indianapolis, IN USA; 5 Department of Crop and Soil Sciences, University of Georgia, Athens, GA USA; 6 Present Address: Department of Chemistry, Mahasarakham University, Mahasarakham, Thailand; 7 Present Address: ZevRoss Spatial Analysis, Ithaca, NY USA

**Keywords:** Element cycles, Geochemistry

Correction to: *Nature Communications* 10.1038/s41467-024-47931-z, published online 18 July 2024

The original version of this Article contained an error in the Abstract, which was previously incorrectly given as ‘dephosphorylarion’. The correct version states ‘dephosphorylation’ in place of ‘dephosphorylarion’. This has been corrected in both the PDF and HTML versions of the Article.

