# Author Correction: Unraveling iron oxides as abiotic catalysts of organic phosphorus recycling in soil and sediment matrices

**DOI:** 10.1038/s41467-024-52106-x

**Published:** 2024-08-30

**Authors:** Jade J. Basinski, Sharon E. Bone, Annaleise R. Klein, Wiriya Thongsomboon, Valerie Mitchell, John T. Shukle, Gregory K. Druschel, Aaron Thompson, Ludmilla Aristilde

**Affiliations:** 1 Department of Civil and Environmental Engineering, Northwestern University, Evanston, IL USA; 2 Stanford Synchrotron Radiation Light Source, SLAC National Accelerator Laboratory, Menlo Park, CA USA; 3 Australian Synchrotron, Australian Nuclear Science and Technology Organisation, Clayton, VIC Australia; 4 Department of Earth Sciences, Indiana University-Purdue University Indianapolis, Indianapolis, IN USA; 5 Department of Crop and Soil Sciences, University of Georgia, Athens, GA USA; 6 Present Address: Department of Chemistry, Mahasarakham University, Mahasarakham, Thailand; 7 Present Address: ZevRoss Spatial Analysis, Ithaca, NY USA

**Keywords:** Element cycles, Geochemistry

Correction to: *Nature Communications* 10.1038/s41467-024-47931-z, published online 18 July 2024

The original version of this Article contained an error in the Abstract, which was previously incorrectly given as ‘ten-fold’. The correct version states ‘twenty-fold’ in place of ‘ten-fold’. This has been corrected in both the PDF and HTML versions of the Article.

