# Supplementary material for: Unraveling iron oxides as abiotic catalysts of organic phosphorus recycling in soil and sediment matrices
Source: Nat Commun. 2024 Jul 18;15:5930. doi: 10.1038/s41467-024-47931-z (PMC11258345; doi:10.1038/s41467-024-47931-z)
Supplement: Supplementary file 1 — Supplementary Information [file 41467_2024_47931_MOESM1_ESM.pdf]

Supplementary Materials for

Unraveling Iron Oxides as Abiotic Catalysts of Organic Phosphorus Recycling in Soil and Sediment Matrices

Jade J. Basinski[1], Annaleise R. Klein[1,2], Sharon E. Bone[3], Wiriya Thongsomboon[1,6], Valerie Mitchell[2], John T. Shukle[4,7], Gregory K. Druschel[4], Aaron Thompson[5], Ludmilla Aristilde[1,*]

[1]Department of Civil and Environmental Engineering, Northwestern University, Evanston, IL, USA.

[2] Australian Synchrotron, Australian Nuclear Science and Technology Organisation, Clayton VIC, Australia.

[3]Stanford Synchrotron Radiation Light Source, SLAC National Accelerator Laboratory, Menlo Park, CA, USA.

[4]Department of Earth Sciences, Indiana University-Purdue University Indianapolis, Indianapolis, IN, USA.

[5]Department of Crop and Soil Sciences, University of Georgia, Athens, GA, USA.

[6]Present address: Department of Chemistry, Mahasarakham University, Mahasarakham, Thailand.

[7]Present address: ZevRoss Spatial Analysis, Ithaca, NY, USA.

*Corresponding author: ludmilla.aristilde@northwestern.edu

The PDF file includes:

Figs. S1 to S17

Tables S1 to S10

SUPPLEMENTAL FIGURES

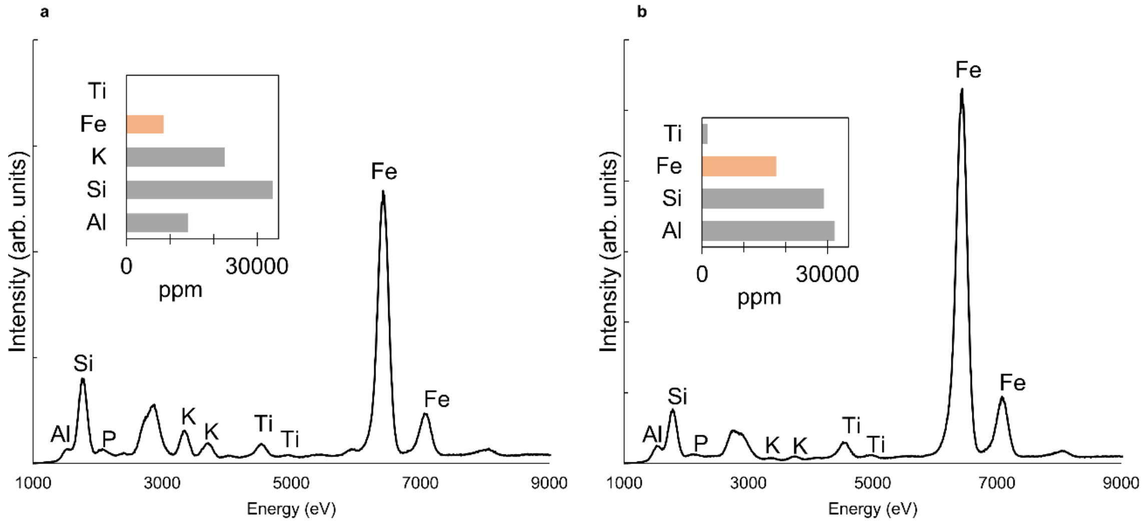

Fig. S1 | Elemental analysis of lake sediment and soil. X-ray fluorescence elemental analysis spectra and quantification (ppm) of the lake sediment (**a**) and the forest soil (**b**). Quantification is shown in the inset bar graph with titanium (Ti), silicon (Si), and aluminum (Al) in gray and iron (Fe) in orange. The y-axis (Intensity) is in arbitrary units (arb. units).

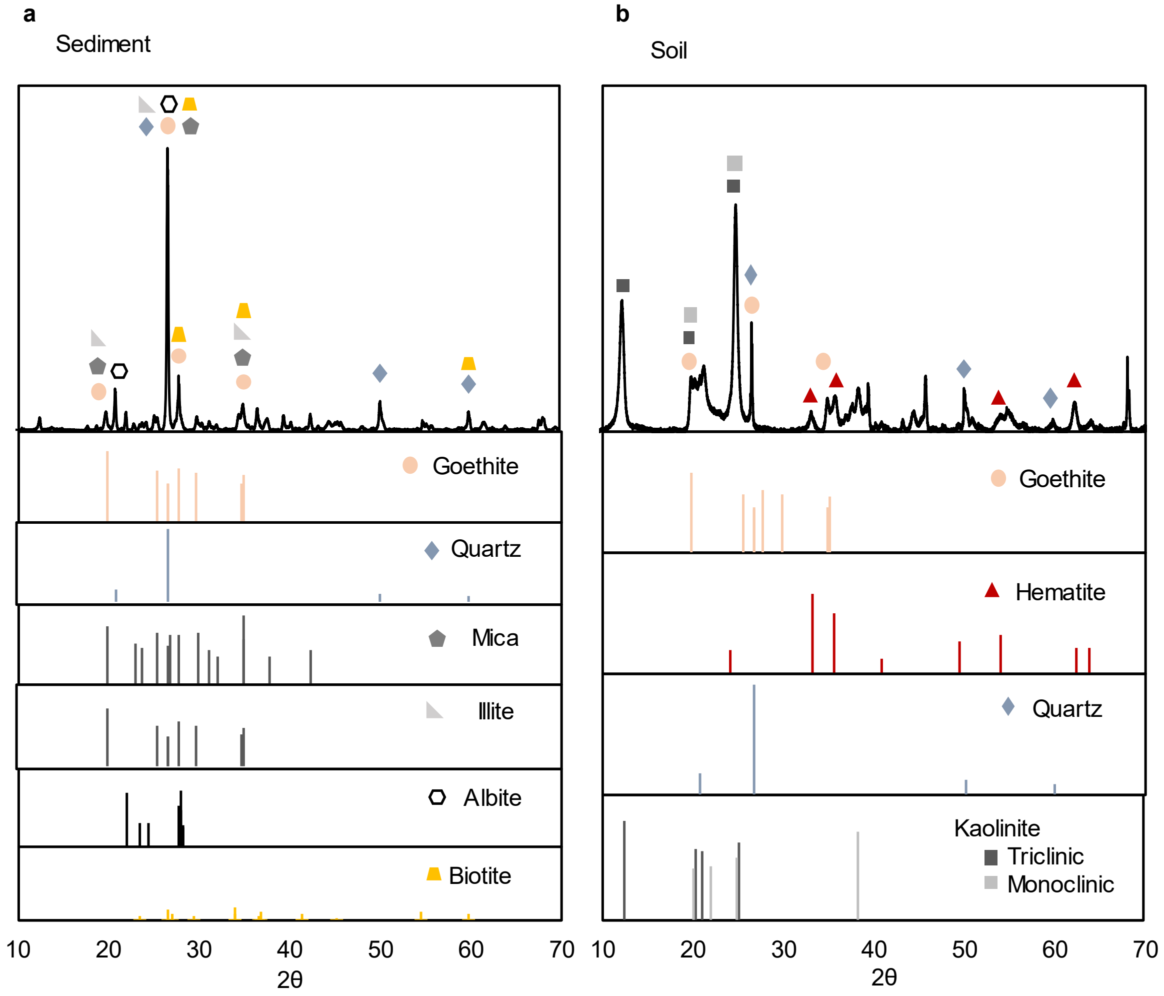

Fig. S2 | Determination of crystalline phases present in lake sediment and soil. X-ray diffraction analysis of crystalline phases in the lake sediment (**a**) and forest soil (**b**). Peak fitting from reference spectra in the Crystallography Open Database was used to identify and quantify the mineral phases present. Goethite is depicted in light orange, hematite in dark red, biotite (Fe-containing mica) in yellow, quartz in light gray, the aluminum silicates (mica, illite, and kaolinite) in dark gray, and the feldspar albite in white.

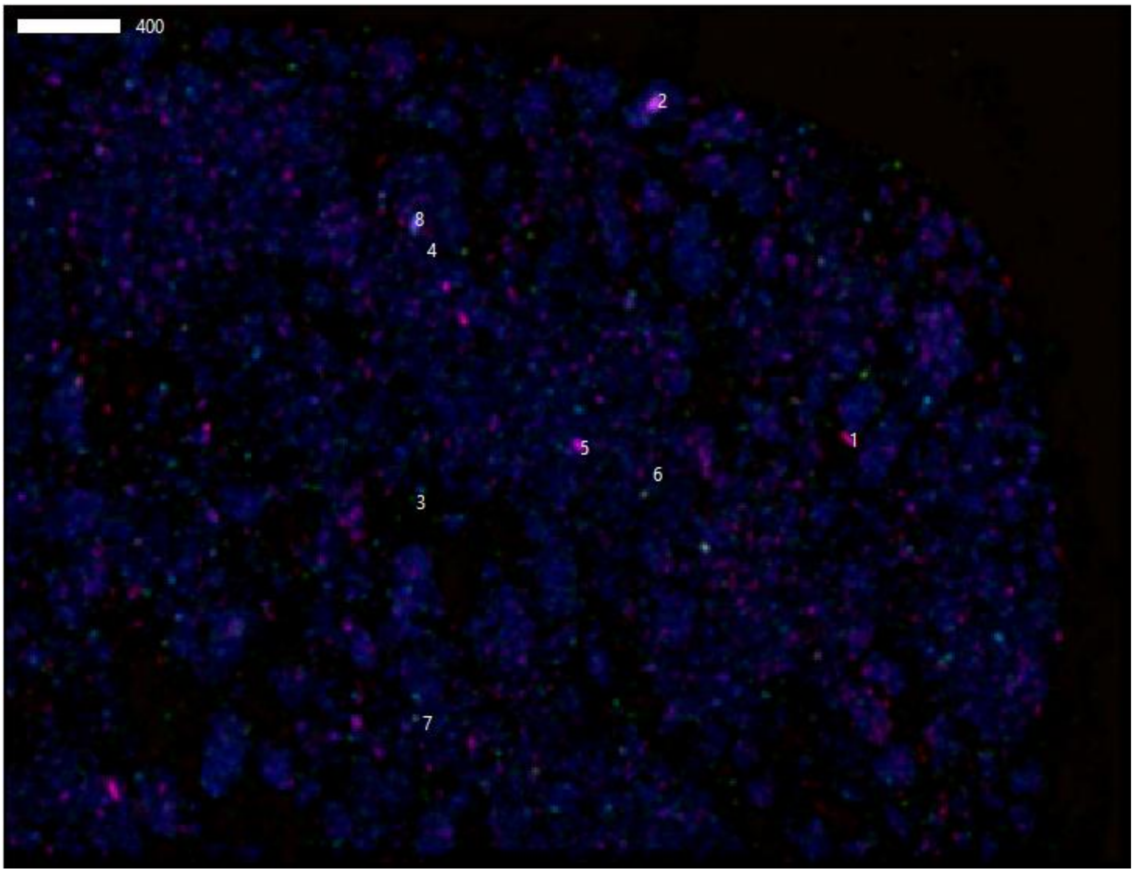

Fig. S3 | Spots chosen for iron (Fe) speciation of sediment sample. Spots were chosen based on principle component analysis (PCA) of Fe data from µ-X-ray fluorescence spectroscopy to determine chemically distinct regions of Fe. PCA component 1 is shown in blue, component 2 in red, and component 3 in green. The white scale bar represents 400 µm. The spots chosen for analysis are numbered in white.

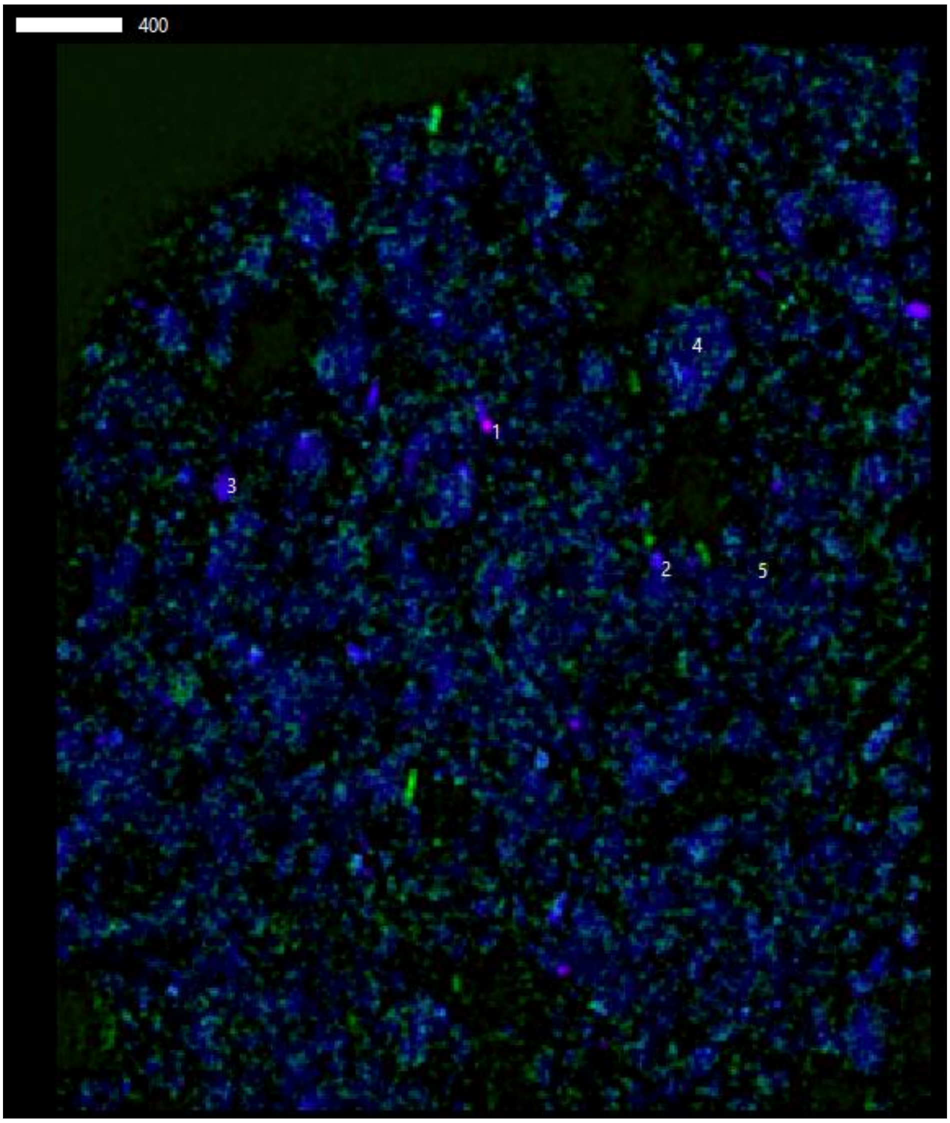

Fig. S4 | Spots chosen for iron (Fe) speciation of soil sample. Spots were chosen based on principle component analysis (PCA) data of Fe from µ-X-ray fluorescence spectroscopy to determine chemically distinct regions of Fe. PCA component 1 is shown in blue, component 2 in red, and component 3 in green. The white scale bar represents 400 µm. The spots chosen for analysis are numbered in white.

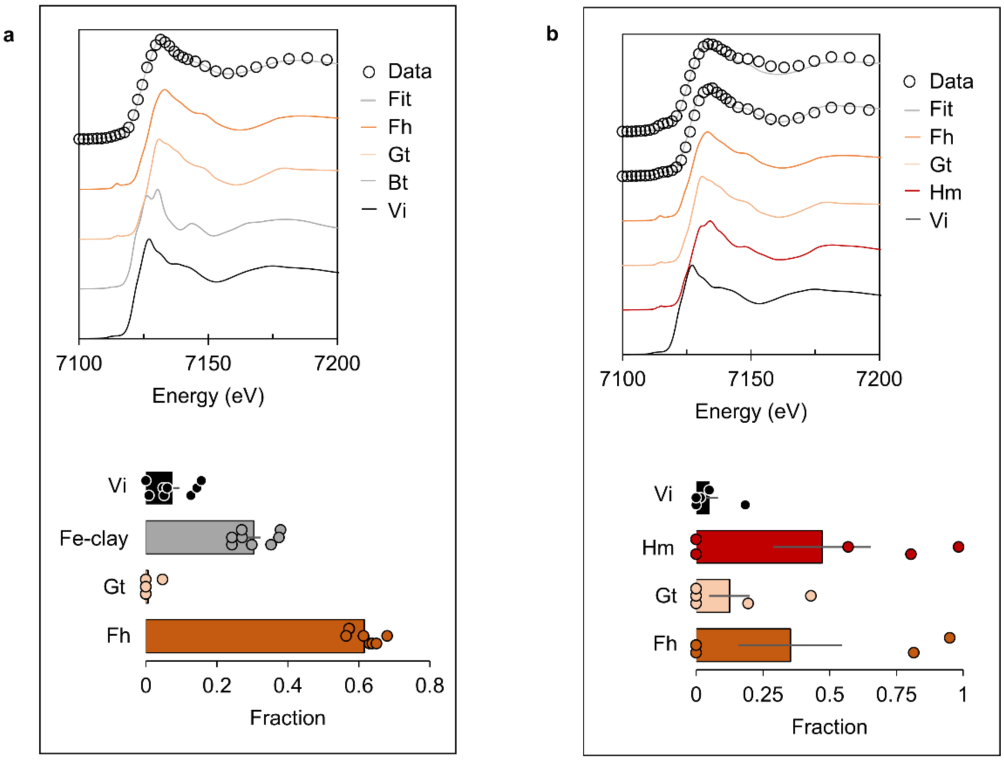

Fig. S5 | Lake sediment and soil characterization of iron (Fe) phases. Fe X-ray absorption near-edge structure (XANES) spectroscopy analysis of the Fe speciation of the **a**, lake sediment from linear combination fitting (LCF) generated from the average of 8 distinct spots. The Fe speciation of the lake sediment from Fe-XANES LCF generated from the average of 5 distinct spots. In **b**, there are two representative Fe-XANES spectra are shown representing the two distinct populations found throughout the soil sample; specifically one high ferrihydrite population (2 spots) and one high hematite population (3 spots). The LCF parameters for each spot are summarized in Supplementary Table 2. Abbreviations used in the figure are as follows: ferrihydrite (Fh; orange), goethite (Gt; light orange), biotite (Bt; biotite), vivianite (Vi; black), and hematite (Hm; dark red). Data is depicted as mean +/- SE.

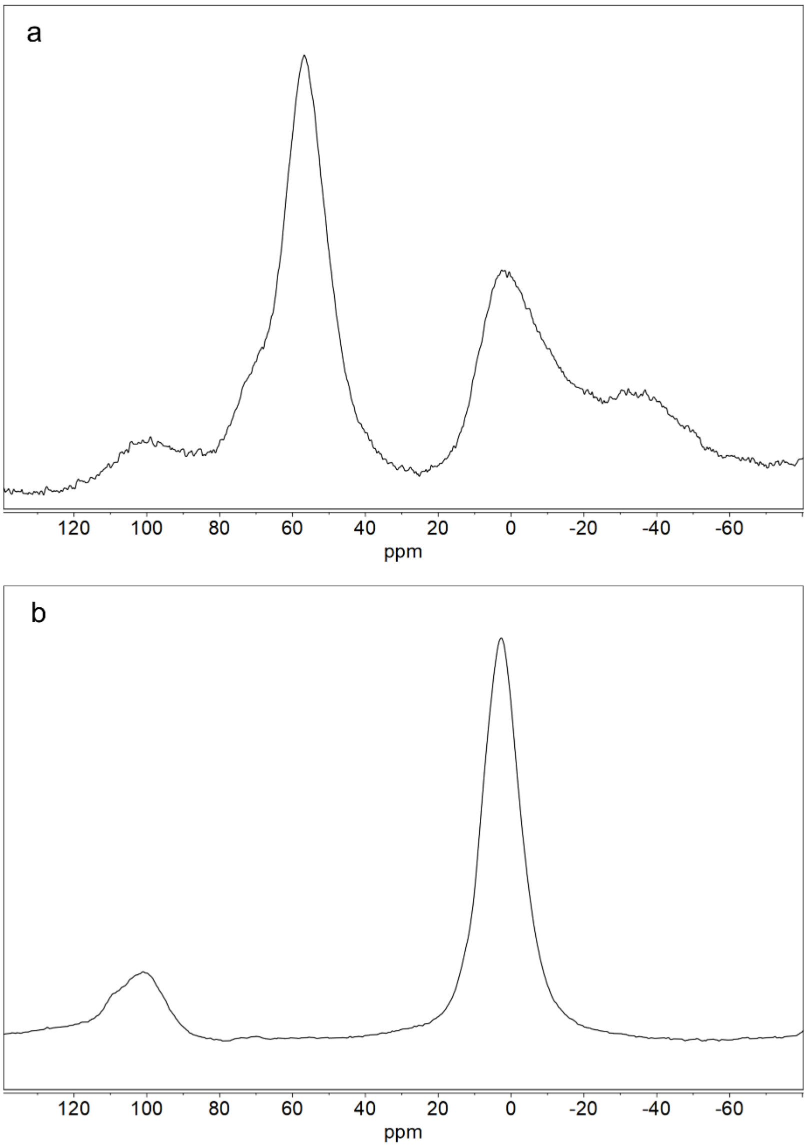

Fig. S6 | ^{27}Aluminum nuclear magnetic resonance (NMR) spectra for sediment and soil samples. ^{27}Aluminum (Al) NMR analysis of the natural samples to determine the presence of amorphous phases of Al. Both the lake sediment (**a**) and the soil sample (**b**) exhibit a peak at ~0 ppm, consistent with Al in the octahedral coordination with oxygen. In addition to octahedral Al, the lake sediment (**a**) exhibits a peak at ~ 60 ppm, consistent with tetrahedral Al. Neither of the samples show the Al (V) peak at ~35 ppm exhibited in reactive, amorphous Al phases[1].

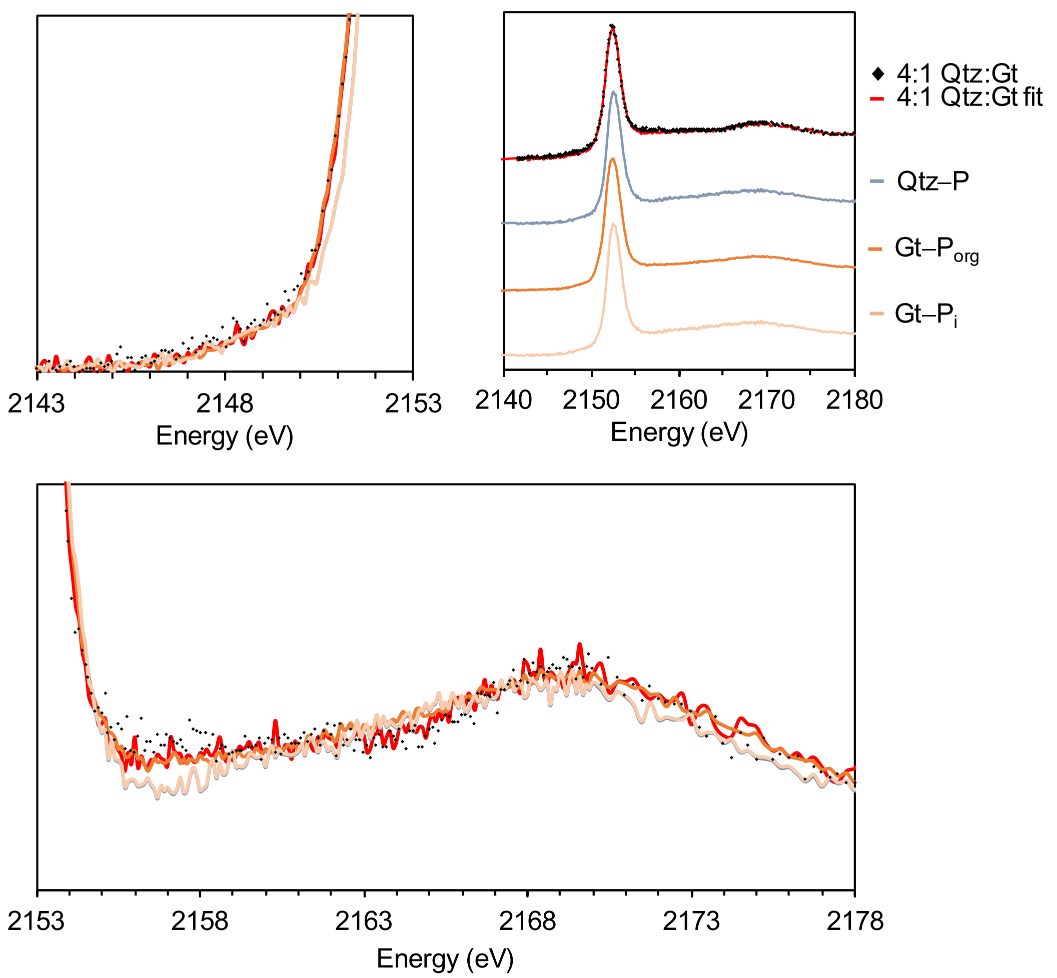

Fig. S7 | Phosphorus (P) K-edge X-ray absorption near-edge structure (XANES) linear combination fits for mixed goethite—quartz system condition. The P K-edge XANES spectra for samples of goethite (Gt) mixed with a second mineral phase, quartz (Qz), in a 1:4 ratio. The data spectrum is depicted by the black diamonds, the linear combination fits by the red lines, and the reference spectra used in the fitting process in gray for Al-silicates and orange for Fe oxides. Inorganic phosphorus bound to Gt (Gt—P$_i$) is shown in light orange and organic phosphorus bound to Gt (Gt—P$_{org}$) is shown in dark orange. The linear combination fitting (LCF) parameters for are summarized in Supplementary Table 3. Spectra of the pre-edge and white line region (2143 – 2153 eV) and the post-edge region (2153 – 2178 eV) are included for clarity.

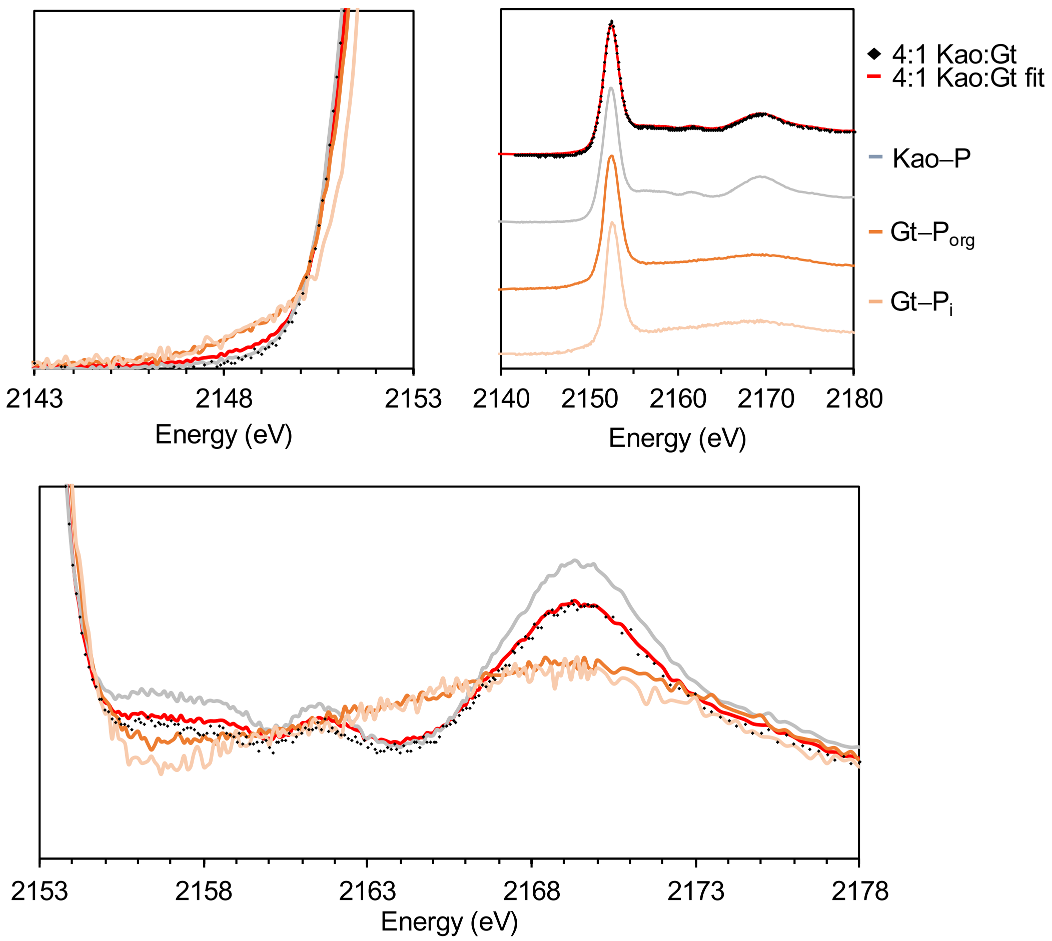

Fig. S8 | Phosphorus (P) K-edge X-ray absorption near-edge structure (XANES) linear combination fits for mixed goethite—kaolinite system condition. The P K-edge XANES spectra for samples of goethite (Gt) mixed with a second mineral phase, kaolinite (Kao), in a 1:4 ratio. The data spectrum is depicted by the black diamonds, the linear combination fits by the red lines, and the reference spectra used in the fitting process in gray for Al-silicates and orange for Fe oxides. Inorganic phosphorus bound to Gt (Gt—P$_i$) is shown in light orange and organic phosphorus bound to Gt (Gt—P$_{org}$) is shown in dark orange. The linear combination fitting parameters for are summarized in Supplementary Table 3. Spectra of the pre-edge and white line region (2143 – 2153 eV) and the post-edge region (2153 – 2178 eV) are included for clarity.

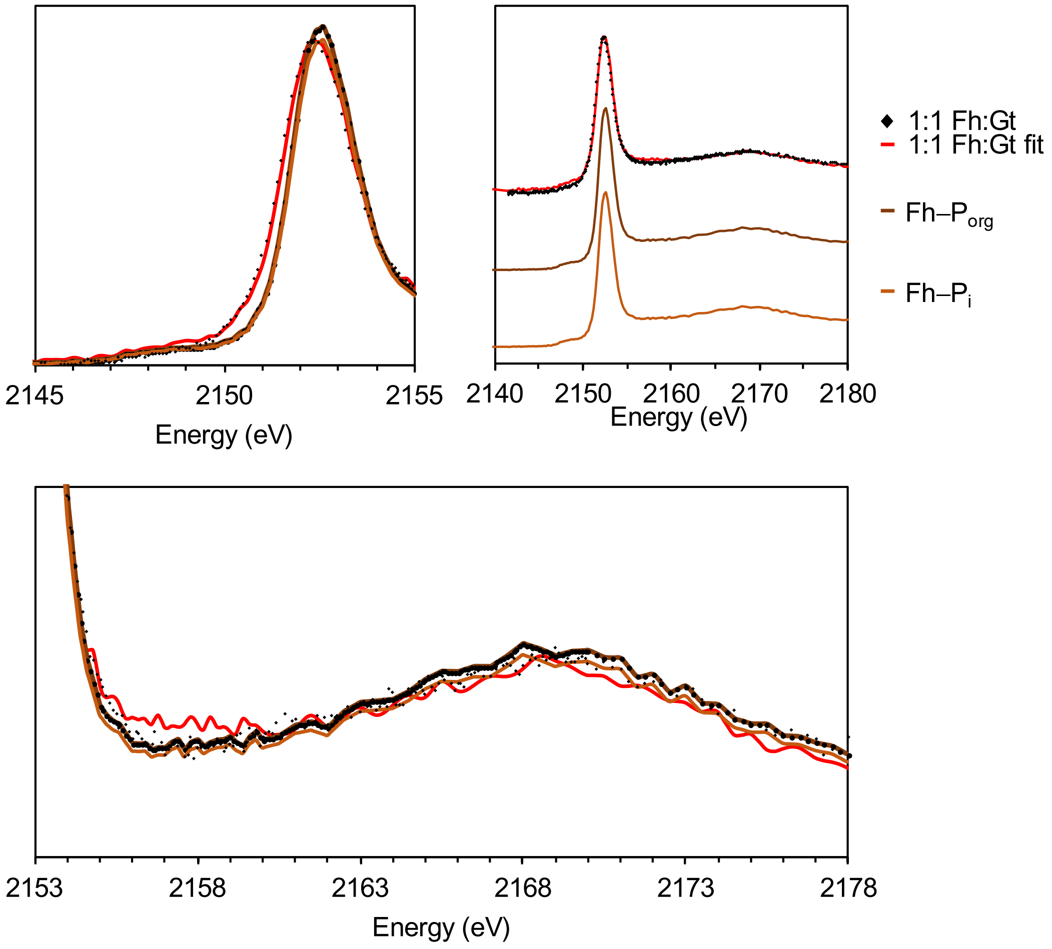

Fig. S9 | Phosphorus K-edge XANES linear combination fits for mixed goethite—ferrihydrite 1:1 system condition. The P K-edge XANES spectra for samples of goethite (Gt) mixed with a second mineral phase, ferrihydrite (Fh) in a 1:1 ratio. The data spectrum is depicted by the black diamonds, the linear combination fits by the red lines, and the reference spectra used in the fitting process in brown for Fe oxides. Inorganic phosphorus bound to Fh (Fh—P$_{i}$) is shown in light brown and organic phosphorus bound to Fh (Fh—P$_{org}$) is shown in dark brown. The linear combination fitting parameters for are summarized in Supplementary Table 3. Spectra of the pre-edge and white line region (2143 – 2153 eV) and the post-edge region (2153 – 2178 eV) are included for clarity.

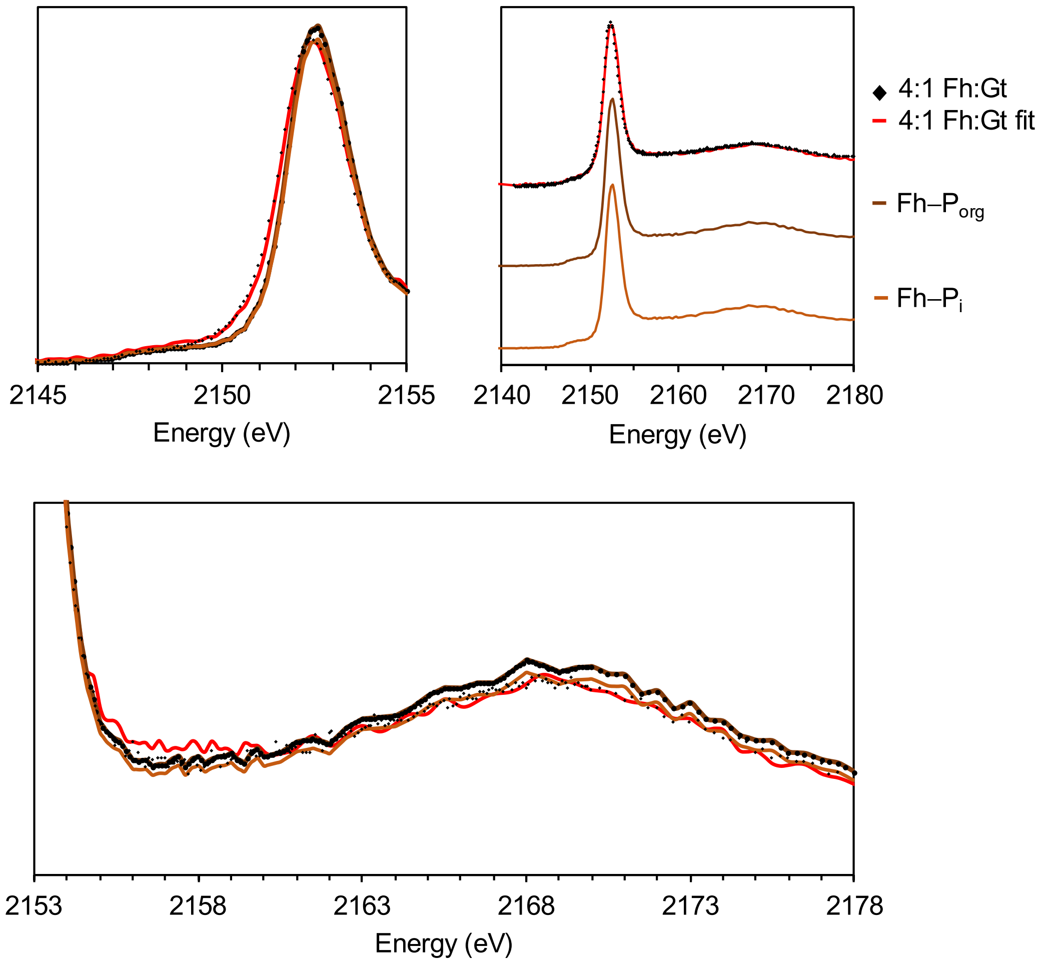

Fig. S10 | Phosphorus (P) K-edge X-ray absorption near-edge structure (XANES) linear combination fits for mixed goethite—ferrihydrite 1:4 system condition. The P K-edge XANES spectra for samples of goethite (Gt) mixed with a second mineral phase, ferrihydrite (Fh) in a 1:4 ratio. The data spectrum is depicted by the black diamonds, the linear combination fits by the red lines, and the reference spectra used in the fitting process in brown for Fe oxides. Inorganic phosphorus bound to Fh (Fh—P_i) is shown in light brown and organic phosphorus bound to Fh (Fh—P_{org}) is shown in dark brown. The linear combination fitting parameters for are summarized in Supplementary Table 3. Spectra of the pre-edge and white line region (2143 – 2153 eV) and the post-edge region (2153 – 2178 eV) are included for clarity.

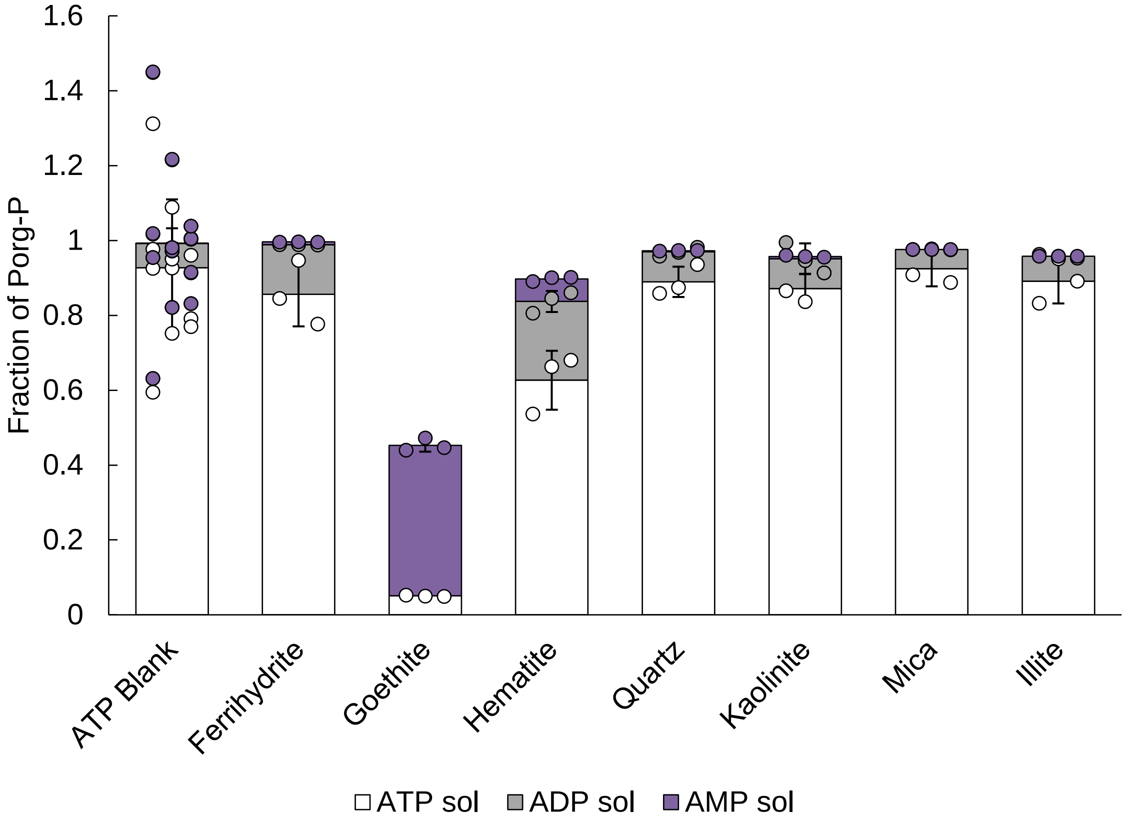

Fig. S11 | Accumulation of organic phosphorus (P_{org}) byproducts in solution after 7 d reactions with adenosine triphosphate (ATP). Quantification of solution P_{org} compounds (ATP; white, adenosine diphosphate, ADP; gray, and adenosine monophosphate, AMP; purple) using liquid-chromatography mass spectrometry analysis. Data are depicted as mean values +/- SD, with error bars representing one standard deviation of n = 12 samples (ATP blank) and n = 3 samples (all other samples).

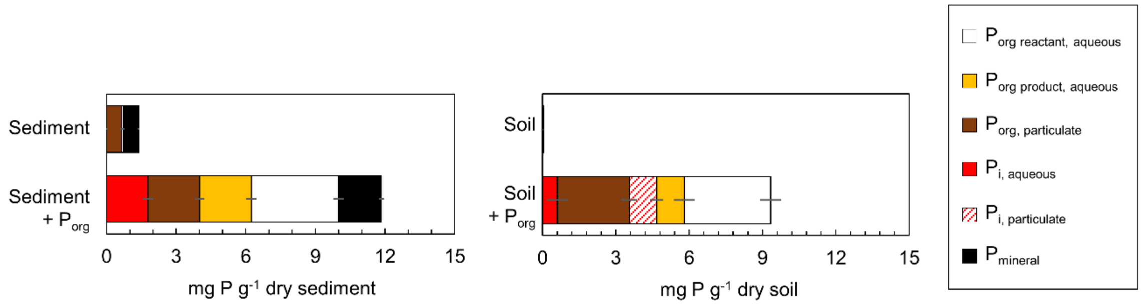

Fig. S12 | Speciation of ribonucleotide-derived phosphorus (P) after reactions with the soil and sediment samples. Aqueous and particulate P species before and after 7 d reaction of adenosine triphosphate (ATP)-P (300 µM or 9.3 mg L^{-1}) with the (left) soil and (right) sediment samples: dissolved P_{org} reactant ($P_{org.\ reactant,\ aqueous}$; white), dissolved P_{org} product ($P_{org\ product,\ aqueous}$; yellow), particulate P_{org} ($P_{org,\ particulate}$; brown), dissolved P_i ($P_{i,\ aqueous}$; red), particulate P_i ($P_{i,\ particulate}$; red stripes), mineral P ($P_{mineral}$; black). Note that the fraction of $P_{org\ product,\ aqueous}$ includes adenosine diphosphate (ADP) and adenosine monophosphate (AMP). Error bars depict the error of fit from linear combination fitting of P K-edge X-ray absorption near-edge structure analysis.

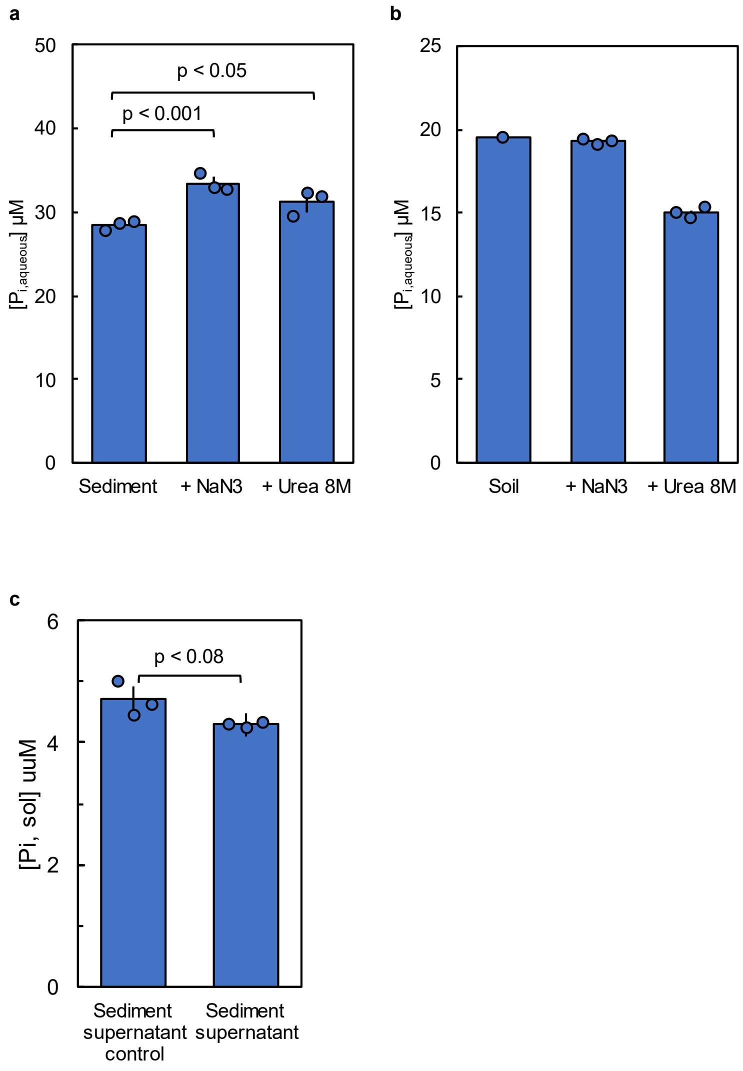

Fig. S13 | Abiotic controls of natural sample reactivity. Quantification of solution inorganic phosphorus (P_i) after reaction of organic phosphorus (P_{org}), 100 µM adenosine triphosphate (ATP), with natural samples without and with the addition of an antimicrobial agent (sodium azide, NaN_3) or a protein denaturing agent (urea 8 M). Results of the abiotic control experiments for the sediment are shown in **a** and results for the soil are shown in **b**. Enzyme extraction experiments were conducted for the sediment sample are shown in **c**. For the enzyme extraction data, the P_i measured in solution of the ATP control replicates were subtracted from the P_i measured after incubation of the sediment sample supernatant with 50 µM ATP for 7 days. For all experiments, n = 3 independent samples and the data are depicted as the mean +/- SD.

Abiotic contribution of solution P_i generation was calculated for each sample by the following equation:

$$\text{Eq. 1}\ Reaction_{Nat\ sample+ATP} = Total = A + M + E;$$

Where Total = Total $P_{i\ measured}$, A = Abiotic, M = Microbial, and E = Enzyme

$$\text{Eq. 2}\ P_{i,NaN3} = Total - M$$

$$\text{Eq. 3}\ P_{i,Urea} = Total - E$$

By combining Eq. 2 and Eq. 3

$$\text{Eq. 4}\ P_{i,NaN3} + P_{i,Urea} = 2\,Total - (M + E)$$

By combining Eq. 1 and Eq. 4

$$\text{Eq. 5}\ P_{i,NaN3} + P_{i,Urea} = 2\,Total - (Total - A)$$

Rearranging Eq. 5

$$\text{Eq. 6}\ A = P_{i,NaN3} + P_{i,Urea} - Total$$

Finally, we get the equation

$$\text{Eq. 7}\ \%\,Abiotic\ Contribution : \frac{P_{i,NaN3} + P_{i,Urea} - Total}{Total}$$

$$Abiotic\ contribution = \frac{NaN3 + Urea - Control - 2(ATP_{control} - Urea_{control})}{Control}$$

which resulted in abiotic contributions for the natural samples ranging between 74% and 100%.

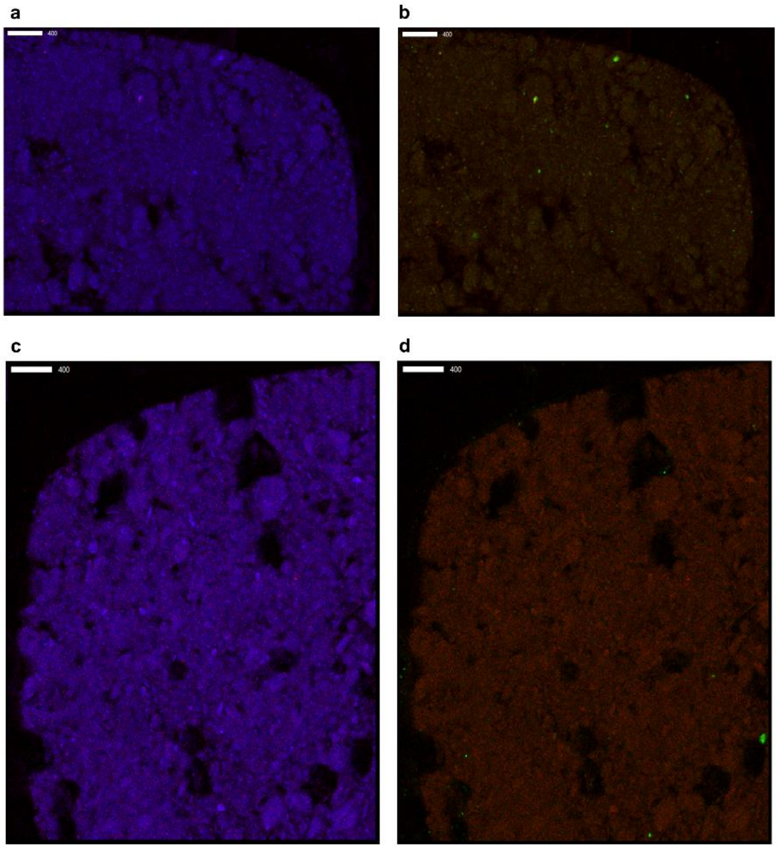

Fig. S14 | Elemental imaging from µ-XANES in environmental samples. Correlation mapping of P (red) with other relevant elements in the sediment (**a**, **b**) and in the soil (**c**, **d**). Fe is shown in blue (**a, c**) and Ca in green (**b**, **d**). In the sediment and soil, the correlation of P with Fe is shown throughout both samples (purple) while P does not seem strongly correlated to Ca. The white scale bar represents 400 µm.

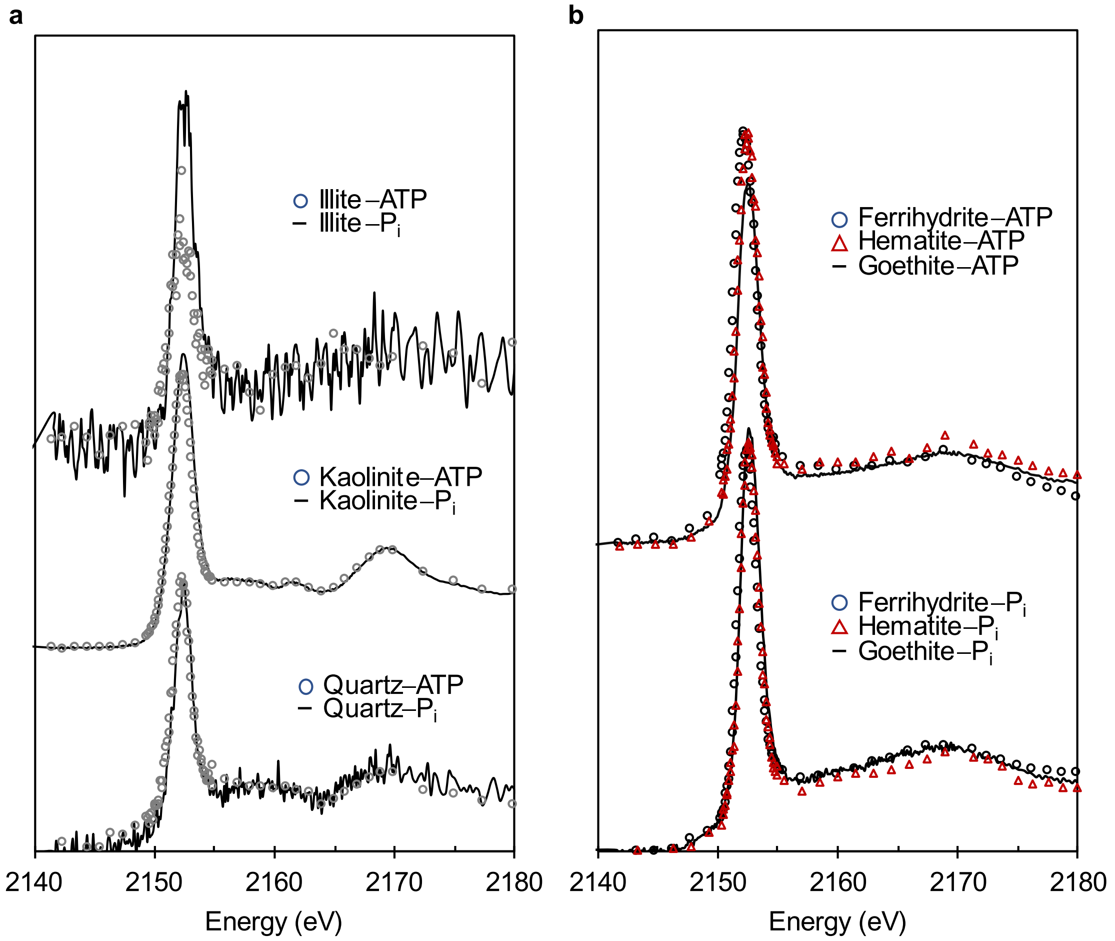

Fig. S15 | Similarities and distinctions in phosphorus (P) K-edge X-ray absorption near-edge structure (XANES) spectra. a, Overlaps of XANES spectra of samples containing illite with adsorbed inorganic P (P_i) and adsorbed organic P (P_{org}) (top), kaolinite with adsorbed P_i and adsorbed P_{org} (middle), or quartz with adsorbed P_i and adsorbed P_{org} (bottom). **b,** Overlaps of XANES spectra of ferrihydrite, hematite, and goethite with (top) P_{org} or (bottom) P_i. In **a,** data for samples with adsorbed P_{org} and adsorbed P_i are shown with circles and black lines, respectively. In **b,** data for samples containing ferrihydrite, hematite, and goethite are shown with black circles, red triangles, black lines, respectively. In **a,** and **b,** P_{org} reactant was adenosine triphosphate (ATP).

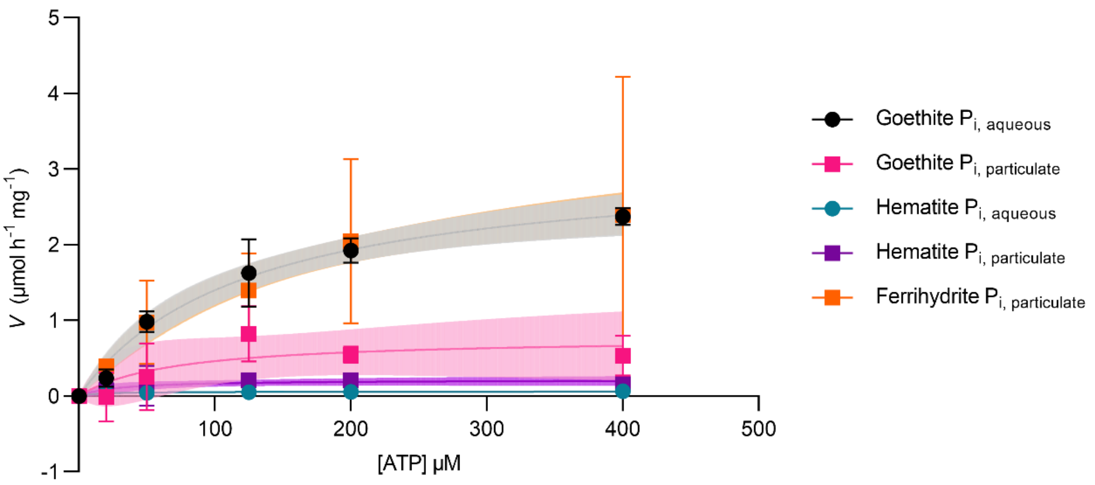

Fig. S16 | Michaelis-Menten kinetics of iron (Fe) oxide-mediated dephosphorylation. Rates of dephosphorylation as determined by concentration of inorganic phosphorus (P_i) plotted as a function of adenosine triphosphate (ATP) added with goethite, hematite, and ferrihydrite. Measured rates of generation for solution P_i (circular data points) and particulate P_i (square data points) are shown separately. Solution P_i was measured by ultraviolet-visible adsorption spectroscopy and the particulate P_i was measured using a combination of mass balance of dephosphorylation products in solution and phosphorus (P) K-edge X-ray absorption near-edge structure (XANES) spectroscopy. The data points represent the rate of P_i generation at each concentration of ATP and the error bars reflect the 95% confidence interval in the rate of P_i generation at each ATP concentration (aqueous) and the combination of standard deviation in rate and error in P K-edge XANES linear combination fitting method (particulate). The shaded region depicts the 95% confidence interval for the model. Goethite solution reactivity is shown in black and goethite adsorption reactivity is shown in pink. Hematite solution reactivity is shown in turquoise and hematite adsorption reactivity is shown in purple. Ferrihydrite adsorption reactivity is shown in orange and no solution reactivity was detected. Model fit parameters are listed in **SI, Table S9**.

a

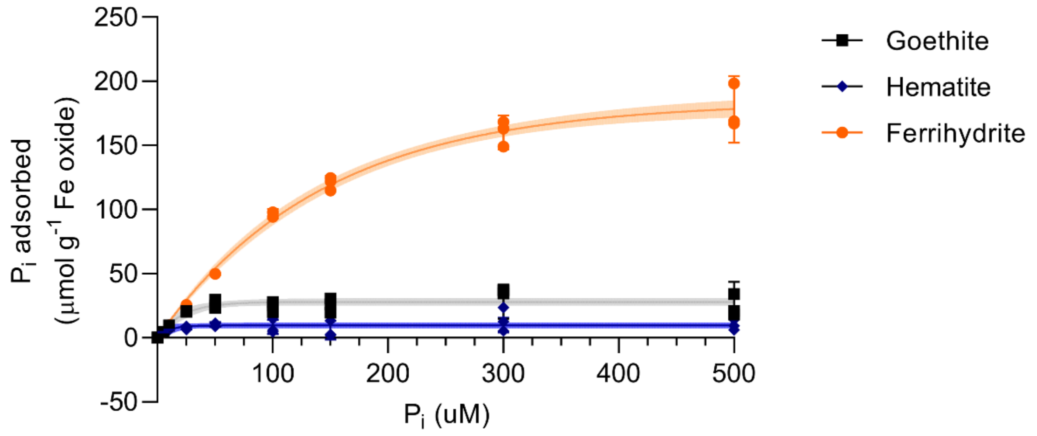

b

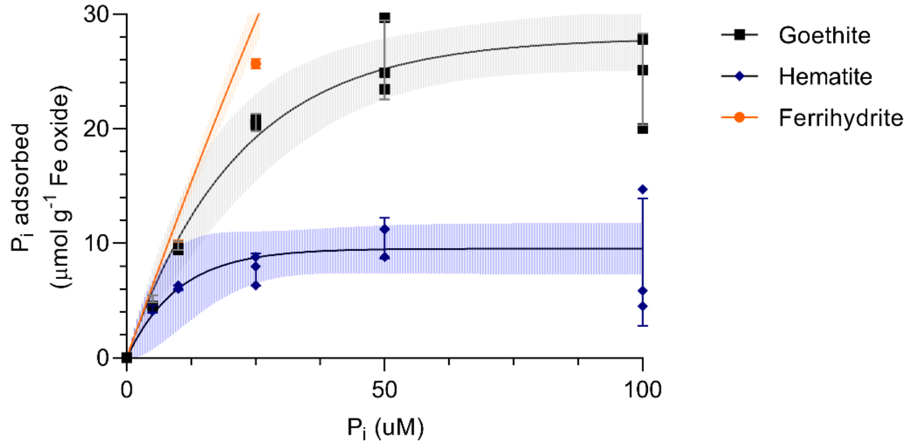

Fig. S17 | Inorganic phosphorus (P_i) adsorption site density of goethite, hematite, and ferrihydrite. Adsorption isotherms were used to determine the total loading of P_i on goethite, hematite, and ferrihydrite. Adsorption isotherms were generated using a 24 h time point. The maximum amount of P_i adsorbed was taken to be the phosphorus (P) site density for each Fe oxide and used to normalize the V_{max} shown in SI Figure S11 to find the turnover number (k_{cat}) for each iron (Fe) oxide. **a,** Isotherms for P_i adsorption onto ferrihydrite, goethite, and hematite. **b**, Zoomed in portions of the adsorption isotherms are shown going to 100 µM P_i in the x-axis and 30 µmol g^{-1} Fe oxide as P_i adsorbed are shown for greater clarity of goethite and hematite adsorption behavior. Error bars represent the standard deviation of 3 replicates. The shaded region depicts the 95% confidence interval of the model fit. Goethite is shown as black squares (data) and a black line (model fit); hematite is shown as purple diamonds (data) and a black line (model fit); and ferrihydrite is shown as orange circles (data) and an orange line (model fit). Model fit parameters are listed in **SI, Table S10**.

SUPPLEMENTAL TABLES

Table S1 | Linear combination fitting parameters for iron (Fe) K-edge X-ray absorption near-edge structure (XANES) spectra of the natural samples. Linear combination fitting parameters used to generate a fitted spectra that matches the sample spectra (shown in Supplementary Fig. 5). Parameters were chosen based on the goodness of fit (R-value < 0.01) and summed to between 0.9 and 1.10, to determine the fraction of Fe-clay (as biotite), vivianite (Fe (II)), and Fe oxides (ferrihydrite (Fh), goethite (Gt), and hematite (Hm)).

Sample	Frac Vivianite[†]	Error	Frac Bt[†]	Error	Frac Hm[‡]	Error	Frac Gt[‡]	Error	Frac Fh[‡]	Error	Sum	R-value	X^2
**Lake Sediment**													
Averaged, Normalized to 1	0.074	0.020	0.304	0.019			0.006	0.006	0.615	0.014	1.00		
Spot 1	0.122	0.02	0.233	0.018					0.606	0.005	0.961	0.001	0.045
Spot 2	0.137	0.031	0.231	0.027					0.585	0.008	0.953	0.003	0.106
Spot 3			0.358	0.007			0.044	0.045	0.54	0.046	0.942	0.003	0.118
Spot 4	0.008	0.036	0.329	0.032					0.595	0.01	0.932	0.004	0.146
Spot 5	0.045	0.038	0.252	0.033					0.632	0.01	0.929	0.004	0.158
Spot 6	0.15	0.027	0.259	0.024					0.548	0.007	0.957	0.002	0.081
Spot 7	0.048	0.032	0.280	0.028					0.609	0.008	0.937	0.003	0.113
Spot 8	0.056		0.350						0.526	0.01	0.932	0.004	0.152
**Forest Soil**													
Averaged, Normalized to 1	0.050	0.031			0.472	0.182	0.125	0.076	0.353	0.194			
Spot 1	0.184	0.011							0.815	0.011	0.999	0.005	0.243
Spot 2	0.018	0.016			1.071	0.016					1.089	0.007	0.461
Spot 3	0.052	0.012							0.987	0.012	1.039	0.005	0.257
Spot 4					0.830	0.101	0.201	0.100			1.031	0.007	0.356
Spot 5					0.591	0.113	0.446	0.113			1.037	0.008	0.445

† Fraction of the normalized spectrum from natural biotite and vivianite samples used in Sutherland *et. al.* 2020[2] and Hansel *et. al.* 2003[3]. These serves as representative spectra for Fe-rich clays and Fe(II) phosphate minerals for linear combination fitting.
‡ Fraction of the normalized spectrum from iron oxides, measured by Sharon E. Bone. This serves as representative spectra for Hm, Gt, and Fh for combination fitting.

Table S2 | Speciation of phosphorus (P) compounds after reaction with minerals and mineral mixtures in replicate measurements. Using different techniques for the quantification of P species after reaction of 50 µM adenosine triphosphate (ATP), adenosine monophosphate (AMP), glucose-6-phosphate (G6P), or phytate with different minerals (1 g L^{-1} or 4 g L^{-1}): $P_{org\ reactant,aqueous}$ (ATP, determined by high-resolution liquid chromatography mass spectrometry), $P_{i,\ aqueous}$ (inorganic phosphorus, determined ultraviolet-visible spectroscopy), $P_{org\ products,aqueous}$ (adenosine diphosphate (ADP) and AMP, determined by high-resolution liquid chromatography mass spectrometry), and $P_{org,\ particulate}$ and $P_{i,\ particulate}$ (determined from mass balance on P from solution species and the linear combination fitting of X-ray absorption near-edge structure spectroscopy data).

* AMP samples were adjusted so the mean of the AMP blank was equivalent to 50 µM AMP-P.

Sample	$P_{i,\ aqueous}$ (µM)	$P_{org,\ particulate}$ (µM)	$P_{i,\ particulate}$ (µM)	$P_{org\ products,\ aqueous}$ (µM)	$P_{org,\ reactant,\ aqueous}$ (µM)
ATP control	2.1	0.0	0.0	7.1	143.0
	1.8	0.0	0.0	4.6	143.6
	1.4	0.0	0.0	4.7	147.6
	1.4	0.0	0.0	5.1	156.2
	2.6	0.0	0.0	5.7	92.3
	4.2	0.0	0.0	10.8	116.7
	3.1	0.0	0.0	9.6	119.5
	2.1	0.0	0.0	6.4	151.7
	2.0	0.0	0.0	7.2	143.8
	1.9	0.0	0.0	6.9	149.1
	1.6	0.0	0.0	7.5	203.6
	1.5	0.0	0.0	8.1	169.1
	1.9	0.0	0.0	9.0	122.9
AMP control*	0.1	0.0	0.0	0.0	47.2
	1.0	0.0	0.0	0.0	44.4
	0.8	0.0	0.0	0.0	45.6
	0.7	0.0	0.0	0.0	47.3
	0.9	0.0	0.0	0.0	49.2
	0.6	0.0	0.0	0.0	53.5
G6P control	1.1	0.0	0.0	0.0	45.8
	0.8	0.0	0.0	0.0	48.2
	0.7	0.0	0.0	0.0	46.9
Phytate control	17.3	0.0	0.0	64.7	309.0
	17.5	0.0	0.0	64.2	306.4
	17.8	0.0	0.0	63.9	305.9
ATP + Mica (1g L^{-1})	3.8	0.0	0.3	8.2	143.2
	3.5	0.0	0.6	8.1	154.1
	4.2	10.7	0.0	8.0	140.0
ATP + Illite (1g L^{-1})	6.4	7.4	0.0	10.8	126.4
	5.6	0.0	0.0	10.1	144.5
	7.2	13.3	0.0	9.3	135.4
ATP + Quartz (1g L^{-1})	2.8	0.0	3.7	11.8	142.3

	3.2	0.0	4.5	13.7	144.9
	3.1	0.0	6.0	16.0	155.0
ATP + Quartz (4g L^{-1})	4.7	10.3	4.1	14.2	181.3
	5.7	47.3	3.2	14.2	112.2
	5.2	0.0	3.9	15.5	140.4
ATP + Kaolinite (1g L^{-1})	4.4	0.0	8.8	21.8	143.1
	4.4	0.0	3.6	13.2	138.2
	4.0	0.0	0.7	7.5	151.1
ATP + Kaolinite (4g L^{-1})	9.1	60.3	6.9	22.1	119.5
	9.2	41.8	7.0	21.6	102.8
	9.3	0.0	10.1	27.3	125.8
ATP + Ferrihydrite (1g L^{-1})	0.7	51.5	18.2	11.1	133.5
	0.6	13.3	4.7	11.3	149.6
	0.6	0.0	0.0	12.1	122.7
ATP + Ferrihydrite (4g L^{-1})	0.2	101.0	36.5	12.5	64.4
	0.5	67.9	24.8	14.8	71.6
	0.4	48.1	21.0	0.0	55.7
ATP + Hematite (1g L^{-1})	11.0	3.8	1.5	25.5	58.8
	11.1	12.0	4.9	30.9	72.7
	11.6	9.4	3.9	32.7	74.6
ATP + Goethite (1g L^{-1})	49.6	28.6	96.3	39.1	1.2
	56.9	18.5	61.6	42.4	1.0
	58.5	8.8	27.9	39.8	0.0
ATP + Quartz (4 g L^{-1}) :Goethite (1g L^{-1})	59.1	126.9	13.8	29.9	1.0
	59.6	106.4	10.0	29.3	0.9
	59.4	55.2	3.3	43.7	1.0
ATP + Kaolinite (4 g L^{-1}):Goethite (1 g L^{-1})	54.6	162.7	6.8	2.0	0.0
	55.2	114.8	4.7	16.0	0.0
	56.8	48.3	1.3	47.4	1.0
ATP + Ferrihydrite (1 g L^{-1}):Goethite (1 g L^{-1})	2.3	210.4	0.0	11.8	1.1
	2.5	172.4	0.0	19.5	1.0
	1.7	122.1	0.0	22.2	1.0
ATP + Ferrihydrite (4 g L^{-1}):Goethite (1g L^{-1})	0.2	200.7	22.2	1.1	1.0
	0.2	169.1	18.7	1.2	0.0
	0.3	126.7	13.9	2.1	0.0
AMP + Mica (1g L^{-1})*	0.6	0.0	0.0	0.0	52.8
	0.6	0.0	0.0	0.0	52.0
	0.7	2.3	0.0	0.0	47.7
AMP + Illite (1g L^{-1})*	0.8	0.0	0.0	0.0	53.1
	0.5	0.0	0.0	0.0	57.0

	0.7	0.0	0.0	0.0	52.0
AMP + Quartz (1g L^{-1})*					
	0.8	1.2	0.0	0.0	48.8
	0.6	0.0	0.0	0.0	51.0
	0.7	0.0	0.0	0.0	52.2
AMP + Kaolinite (1g L^{-1})*					
	1.4	0.0	0.0	0.0	51.2
	1.7	0.0	0.0	0.0	58.8
	1.7	0.0	0.0	0.0	51.0
AMP + Ferrihydrite (1g L^{-1})					
	0.0	6.9	37.3	0.0	2.4
	0.0	6.8	37.0	0.0	2.9
	0.0	6.8	36.8	0.0	3.1
AMP + Hematite (1g L^{-1})*					
	0.0	6.9	37.3	0.0	2.4
	0.0	6.8	37.0	0.0	2.9
	0.0	6.8	36.8	0.0	3.1
AMP + Goethite (1g L^{-1})*	0.2	11.6	4.4	0.0	24.7
	0.2	15.7	6.0	0.0	19.1
	0.3	11.3	4.3	0.0	25.0
G6P + Mica (1g L^{-1})	0.7	2.9	0.0	0.0	44.2
	0.5	5.0	0.0	0.0	42.4
	0.6	6.3	0.0	0.0	41.0
G6P + Illite (1g L^{-1})	0.6	0.0	0.0	0.0	48.2
	0.6	1.9	0.0	0.0	45.3
	0.6	0.0	0.0	0.0	47.4
G6P + Quartz (1g L^{-1})	0.8	0.0	0.0	0.0	47.2
	0.9	0.0	0.0	0.0	47.8
	0.8	1.0	0.0	0.0	46.0
G6P + Kaolinite (1g L^{-1})	1.0	2.8	0.0	0.0	44.0
	0.9	2.5	0.0	0.0	44.5
	1.0	3.8	0.0	0.0	43.0
G6P + Ferrihydrite (1g L^{-1})	0.7	21.9	0.0	0.0	25.3
	0.6	17.7	0.0	0.0	29.6
	0.7	20.2	0.0	0.0	26.9
G6P + Hematite (1g L^{-1})	0.5	2.2	0.0	0.0	45.1
	0.7	0.8	0.0	0.0	46.4
	0.7	0.0	0.0	0.0	47.5
G6P + Goethite (1g L^{-1})	0.7	0.0	0.0	0.0	48.5
	0.7	2.8	0.0	0.0	44.4
	1.1	5.4	0.0	0.0	41.3
Phytate + Mica (1g L^{-1})	17.0	81.6	0.0	52.6	237.8

	17.6	60.5	0.0	55.1	255.7
	17.4	53.9	0.0	55.9	261.7
Phytate + Illite (1g L^{-1})	16.9	117.0	0.0	50.1	204.9
	16.5	119.3	0.0	48.9	204.2
	17.2	120.7	0.0	49.7	201.4
Phytate + Quartz (1g L^{-1})	17.3	12.3	0.0	62.4	296.9
	18.1	10.7	0.0	62.5	297.6
	18.1	4.4	0.0	62.9	303.4
Phytate + Kaolinite (1g L^{-1})	17.9	18.7	0.0	62.1	290.2
	18.1	16.2	0.0	62.1	292.4
	17.9	9.4	0.0	61.4	300.2
Phytate + Ferrihydrite (1g L^{-1})	1.2	29.1	17.4	59.6	281.6
	1.2	35.7	21.2	59.3	271.4
	1.2	19.6	11.7	62.1	294.3
Phytate + Hematite (1g L^{-1})	14.8	50.8	6.8	56.3	260.1
	15.5	46.1	6.2	57.1	264.0
	15.0	44.1	5.9	57.1	266.8
Phytate + Goethite (1g L^{-1})	10.2	87.8	7.3	55.4	228.1
	10.2	92.4	7.7	53.1	225.5
	11.2	97.6	8.1	52.9	219.0

Table S3 | Linear combination fitting parameters for phosphorus (P) K-edge X-ray absorption near-edge structure (XANES) spectra in mixed-mineral and pure mineral experiments. Linear combination fitting (LCF) parameters used to generate a fitted spectra that matches the sample spectra (shown in Supplementary Fig. 5). Parameters were chosen based on the goodness of fit (R-value < 0.01) and summed to between 0.90 and 1.10, to determine the fraction of iron (Fe) oxide-associated inorganic phosphorus (P_i), Fe oxide-associated organic phosphorus (P_{org}), and Al-silicate-associated P. In samples with goethite and ferrihydrite, P is assumed to be associated with the Fe oxide with the greater surface area, ferrihydrite.

† Due to low loading of P in glucose-6-phosphate (G6P) + hematite and G6P + goethite reactions, gaussian smoothing (kernel size of 11 and width of 4) was performed to ensure the noise of the spectra did not interfere with LCF analysis.

***** Due to the presence of P_i in the phytate stock, the fraction of P_{org} spectra calculated by the LCF process includes some percentage of P_i character and was adjusted accordingly in $P_{i,\ particulate}$ data shown in SI **Table S2**. Phytate P_{org} spectra contained 36.4% P_i content for ferrihydrite, 4.5% P_i content for hematite, and 7.0% P_i content for goethite.

Sample	Initial P_{org} Reacted	Frac Fe oxide-P_{org}	Error	Frac Fe oxide-P_i	Error	Frac Al-silicate-P	Error	Sum	R-value	X-squared
Quartz:Goethite 4:1	ATP	0.61	0.03	0.07	0.02	0.29	0.01	0.96	0.005	1.043
Kaolinite:Goethite 4:1	ATP	0.22	0.02	0.06	0.01	0.74	0.01	1.02	0.001	0.320
Ferrihydrite:Goethite 1:1	ATP	0.65	0.02	0.31	0.02			0.97	0.006	1.561
Ferrihydrite:Goethite 4:1	ATP	0.52	0.01	0.44	0.01			0.96	0.003	0.800
Goethite	ATP	0.22	0.01	0.79	0.01			1.00	0.001	0.348
Ferrihydrite	ATP	0.73	0.02	0.27	0.02			1.00	0.004	0.645
Hematite	ATP	0.72	0.02	0.30	0.02			1.02	0.004	1.193
Goethite	AMP	0.73	0.02	0.28	0.02			1.02	0.005	0.82

Ferrihydrite	AMP	0.17	0.03	0.92	0.03			1.09	0.008	2.11
Hematite	AMP	0.54	0.02	0.49	0.02			1.03	0.010	2.07
Goethite	G6P†	1.04	0.01	0.01	0.01			1.05	0.001	0.88
Ferrihydrite	G6P	1.00	0.00	-0.01	0.00			0.99	0.001	0.52
Hematite	G6P†	0.98	0.02	0.07	0.02			1.04	0.001	0.88
Goethite	Phytate*	0.93	0.00	0.05	0.00			0.98	0.000	0.23
Ferrihydrite	Phytate*	1.00	0.00	0.02	0.00			1.02	0.000	0.25
Hematite	Phytate*	0.95	0.01	0.03	0.01			0.98	0.001	0.47

Table S4 | Average and error values for speciation of P compounds after reaction with single minerals and mineral mixtures. Average and standard deviation of P species (listed in μM) replicates listed in Supplementary Table 6. Mineral names are shortened: mica (Mic), illite (Ilt), quartz (Qtz), and kaolinite (Kao). Phytate is shortened to Phy. Abbreviations for iron (Fe) oxides are the same as used in the main text: ferrihydrite (Fh), hematite (Hm), and goethite (Gt).

* AMP samples were adjusted so the mean of the AMP blank was equivalent to 50 μM AMP-P.

Sample	$P_{i,aq}$	Error	$P_{org,ads}$	Error	$P_{i,ads}$	Error	$P_{org,reacted,aq}$	Error	$P_{org,unreacted}$	Error
ATP Control	2.1	0.8	0.0	0.0	0.0	0.0	7.1	1.9	143.0	27.2
ATP + Mic (1g L^{-1})	3.8	0.3	3.6	5.0	0.3	0.2	8.2	0.1	145.8	6.0
ATP + Ilt (1g L^{-1})	6.4	0.6	6.9	5.4	0.0	0.0	10.8	0.6	126.4	7.4
ATP + Qtz (1g L^{-1})	3.0	0.2	0.0	0.0	4.7	0.9	13.8	1.7	147.4	5.5
ATP + Qtz (4g L^{-1})	5.2	0.4	19.2	20.3	3.7	0.4	14.6	0.6	144.6	28.4
ATP + Kao (1g L^{-1})	4.3	0.2	0.0	0.0	4.4	3.3	14.2	5.9	144.1	5.3
ATP + Kao (4g L^{-1})	9.2	0.1	34.0	25.2	8.0	1.5	23.6	2.6	116.1	9.7
ATP + Fh (1g L^{-1})	0.6	0.1	21.6	21.8	7.6	7.7	11.5	0.4	135.3	11.1
ATP + Fh (4g L^{-1})	0.3	0.1	72.3	21.9	27.4	6.6	9.1	6.5	63.9	6.5
ATP + Hm (1g L^{-1})	11.2	0.2	8.4	3.4	3.4	1.4	29.7	3.1	68.7	7.0
ATP + Gt (1g L^{-1})	55.0	3.8	18.6	8.1	61.9	27.9	40.4	1.4	0.8	0.5
ATP + Qtz:Gt 4:1	59.4	0.2	96.2	30.1	9.0	4.3	34.3	6.7	1.0	0.0
ATP + Kao:Gt 4:1	55.5	1.0	108.6	46.9	4.3	2.3	21.8	19.0	0.3	0.5
ATP + Fh:Gt 1:1	2.2	0.4	168.3	36.2	0.0	0.0	17.8	4.4	1.0	0.0

ATP + Fh:Gt 1:1	0.2	0.0	165.5	30.3	18.3	3.4	1.5	0.4	0.3	0.5
AMP Control*	0.7	0.3	0.0	0.0	0.0	0.0	0.0	0.0	47.9	2.9
AMP + Mic (1g L^{-1})*	0.6	0.0	0.8	1.1	0.0	0.0	0.0	0.0	50.9	2.2
AMP + Ilt (1g L^{-1})*	0.7	0.1	0.0	0.0	0.0	0.0	0.0	0.0	54.0	2.6
AMP + Qtz (1g L^{-1})*	0.7	0.1	0.4	0.5	0.0	0.0	0.0	0.0	50.7	1.4
AMP + Kao (1g L^{-1})*	1.6	0.1	0.0	0.0	0.0	0.0	0.0	0.0	53.7	1.9
AMP + Fh (1g L^{-1})*	0.0	0.0	6.8	0.0	36.8	0.2	0.0	0.0	2.8	0.3
AMP + Hm (1g L^{-1})*	0.0	0.1	0.0	0.0	0.0	0.0	0.0	0.0	48.6	1.0
AMP + Gt (1g L^{-1})*	0.0	0.0	5.8	0.4	2.2	0.1	0.0	0.0	38.3	0.5
G6P Control*	0.9	0.2	0.0	0.0	0.0	0.0	0.0	0.0	46.9	1.0
G6P + Mic (1g L^{-1})	0.6	0.1	4.7	1.6	0.0	0.0	0.0	0.0	42.5	1.3
G6P + Ilt (1g L^{-1})	0.6	0.0	0.6	1.6	0.0	0.0	0.0	0.0	47.0	1.2
G6P + Qtz (1g L^{-1})	0.9	0.3	0.0	0.0	0.0	0.0	0.0	0.0	47.0	0.8
G6P + Kao (1g L^{-1})	1.0	0.0	3.0	1.2	0.0	0.0	0.0	0.0	43.8	0.6

G6P + Fh (1g L^{-1})	0.8	0.0	15.0	2.0	0.0	0.0	0.0	0.0	32.1	1.8
G6P + Hm (1g L^{-1})	0.6	0.1	1.0	1.4	0.0	0.0	0.0	0.0	46.3	1.0
G6P + Gt (1g L^{-1})	0.8	0.2	2.7	3.1	0.0	0.0	0.0	0.0	44.7	2.9
Phy Control	17.5	0.2	0.0	0.0	0.0	0.0	64.2	0.3	307.1	1.4
Phy + Mic (1g L^{-1})	17.3	0.2	65.3	11.8	0.0	0.0	54.5	1.4	251.7	10.2
Phy + Ilt (1g L^{-1})	16.9	0.3	119.0	1.5	0.0	0.0	49.5	0.5	203.5	1.5
Phy + Qtz (1g L^{-1})	17.9	0.4	9.1	3.4	0.0	0.0	62.6	0.2	299.3	2.9
Phy + Kao (1g L^{-1})	18.0	0.1	14.8	3.9	0.0	0.0	61.9	0.3	294.3	4.3
Phy + Fh (1g L^{-1})	1.2	0.0	28.1	6.6	16.8	3.9	60.3	1.2	282.5	9.4
Phy + Hm (1g L^{-1})	15.1	0.3	47.0	2.8	6.3	0.4	56.8	0.4	263.6	2.7
Phy + Gt (1g L^{-1})	10.6	0.5	92.6	4.0	7.7	0.3	53.8	1.1	224.2	3.8

Table S5 | Linear combination fitting parameters for phosphorus (P) K-edge X-ray absorption near-edge structure (XANES) spectra for sediment. Linear combination fitting (LCF) parameters used to generate a fitted spectra that matches the sample spectra (shown in Supplemental Fig. 1). Parameters were chosen based on the goodness of fit (R-value < 0.03) and summed to between 0.85 and 1.15, to determine the fraction of calcium (Ca) phosphate mineral (Ca-P_i), iron phosphate mineral clusters (Fe-P_i), Fe oxide-associated organic phosphorus (P_{org}) as ferrihydrite-associated P_{org} (Fh-P_{org}) and goethite-associated P_{org} (Gt-P_{org}), and silicate-associated P as quartz-associated P (Qtz-P) and illite-associated P (Ilt-P).

Sample	Frac Fh-P_{org}	Error	Frac Gt-P_{org}	Error	Frac Fe-P_i	Error	Frac Ca-P_i	Error	Frac Qtz-P	Error	Frac Ilt-P	Error	Sum	R-value	X-squared
Sediment	0.22	0.01			0.44	0.02	0.10	0.02	0.18	0.02	0.02	0.01	0.96	0.006	1.107
Sediment + ATP	0.15	0.05	0.13	0.11	0.40	0.03	0.06	0.02	0.21	0.02	0.01	0.01	0.96	0.006	1.201

Table S6 | Linear combination fitting parameters for phosphorus (P) K-edge X-ray absorption near-edge structure (XANES) spectra for the soil. Linear combination fitting (LCF) parameters used to generate a fitted spectra that matches the sample spectra (shown in Supplemental Fig. 1). Parameters were chosen based on the goodness of fit (R-value < 0.03) and summed to between 0.85 and 1.15, to determine the fraction of iron (Fe) oxide-associated inorganic phosphorus (P_i) as hematite-associated P_i (Hm-P_i), Fe oxide-associated organic phosphorus (P_{org}) as ferrihydrite-associated P_{org} (Fh-P_{org}) and hematite-associated P_{org} (Hm-P_{org}), and aluminosilicate-associated P as kaolinite-associated P (Kao-P).

Sample	Frac Fh-P_{org}	Error	Frac Hm-P_{org}	Error	Frac Hm-P_i	Error	Frac Kao-P	Error	Sum	R-value	X-squared
Soil	0.39	0.05			0.51	0.07			0.90	0.029	9.618
Soil + ATP	0.29	0.03	0.38	0.07	0.27	0.07	0.04	0.03	0.99	0.006	1.916

Table S7 | Normalized linear combination fitting parameters for phosphorus (P) K-edge X-ray absorption near-edge structure (XANES) spectra for the natural samples. Normalization of the linear combination fitting (LCF) parameters for the sediment and soil samples (Supplemental Table 5 and Supplemental Table 6). The normalized parameters were then grouped into the following categories: iron (Fe) oxide-associated inorganic phosphorus (P_i), Fe oxide-associated organic phosphorus (P_{org}), aluminum (Al)-silicate-associated phosphorus (P), and mineral P (in the form of calcium phosphate and Fe(II) phosphate).

Sample	Frac Fe oxide-P_i	Error	Frac Fe oxide-P_{org}	Error	Frac Al-silicate-P	Error	Frac Mineral P	Error
Sediment			0.23	0.01	0.21	0.02	0.56	0.03
Sediment + ATP			0.30	0.12	0.23	0.02	0.47	0.03
Soil	0.56	0.08	0.44	0.05				
Soil + ATP	0.28	0.07	0.68	0.07	0.04	0.03		

Table S8 | Mineral speciation and Ca content in natural samples. Mineral speciation was determined by fitting the X-ray diffraction (XRD) patterns of reference minerals (crystalline phase) and iron (Fe) K-edge X-ray absorption near-edge structure (XANES) linear combination fitting (amorphous and semi-crystalline phases, fitting shown in Supplementary Fig. 5). Content of Ca in each natural samples were determined through extraction (aqua regia for sediment and Mehlich for soil).

	Fractional contribution by mass determined by XRD and Fe XANES fitting								Extraction
	Quartz	Clays	Micas	Feldspars	Hematite	Goethite	Ferrihydrite	Vivianite	Ca (ppm)
Sediment	0.21	0.16	0.34	0.16		0.02	0.10	0.01	4198.2
Soil	0.12	0.69			0.04	0.04	0.10	0.00	351.5

Table S9 | Michaelis-Menten model fitting parameters for iron (Fe) oxide-mediated dephosphorylation. Model fit for V_{max} including confidence intervals, degrees of freedom, and effect size for curve fitting.

		95% Confidence Interval			
	Model fit (V_{max})	Lower limit	Upper limit	R^2	Degrees of Freedom
Goethite $P_{i,aqueous}$	3.383	2.698	4.645	0.97	4
Goethite $P_{i,particulate}$	0.7468	0.4545	1.7	0.88	2
Hematite $P_{i,aqueous}$	0.06439	0.0547	0.07657	0.73	4
Hematite $P_{i,particulate}$	0.272	0.1635	0.5759	0.59	3
Ferrihydrite $P_{i,particulate}$	3.218	2.585	4.241	0.98	3

Table S10 | Isotherm model fitting for Fe oxide mineral P_i site density. Model fit for adsorption isotherms P_i site density including confidence intervals, degrees of freedom, and effect size for curve fitting.

		95% Confidence Interval			
	Model fit (P_i density)	Lower limit	Upper limit	R^2	Degrees of Freedom
Goethite	25.99	25.99	28.66	0.94	14
Hematite	9.096	9.096	11.29	0.56	14
Ferrihydrite	184.1	172.4	197.3	0.98	23

Supplementary References

1. Lee, S. K., Lee, S. B., Park, S. Y., Yi, Y. S. & Ahn, C. W. Structure of Amorphous Aluminum Oxide. *Phys. Rev. Lett.* **103**, 095501 (2009).
2. Sutherland, K. M., Wankel, S. D., Hein, J. R. & Hansel, C. M. Spectroscopic Insights Into Ferromanganese Crust Formation and Diagenesis. *Geochemistry, Geophysics, Geosystems* **21**, e2020GC009074 (2020).
3. Hansel, C. M. *et al.* Secondary mineralization pathways induced by dissimilatory iron reduction of ferrihydrite under advective flow. *Geochimica et Cosmochimica Acta* **67**, 2977–2992 (2003).
