# Supplementary material for: Unraveling iron oxides as abiotic catalysts of organic phosphorus recycling in soil and sediment matrices
Source: Nat Commun. 2024 Jul 18;15:5930. doi: 10.1038/s41467-024-47931-z (PMC11258345; doi:10.1038/s41467-024-47931-z)
Supplement: Supplementary file 3 — Reporting Summary [file 41467_2024_47931_MOESM3_ESM.pdf]

nature portfolio

Corresponding author(s): Dr. Ludmilla Aristilde

Last updated by author(s): March 26, 2024

Reporting Summary

Nature Portfolio wishes to improve the reproducibility of the work that we publish. This form provides structure for consistency and transparency in reporting. For further information on Nature Portfolio policies, see our Editorial Policies and the Editorial Policy Checklist.

Statistics

For all statistical analyses, confirm that the following items are present in the figure legend, table legend, main text, or Methods section.

n/a	Confirmed	
☐	☒	The exact sample size (*n*) for each experimental group/condition, given as a discrete number and unit of measurement
☐	☒	A statement on whether measurements were taken from distinct samples or whether the same sample was measured repeatedly
☐	☒	The statistical test(s) used AND whether they are one- or two-sided<br>*Only common tests should be described solely by name; describe more complex techniques in the Methods section.*
☒	☐	A description of all covariates tested
☐	☒	A description of any assumptions or corrections, such as tests of normality and adjustment for multiple comparisons
☐	☒	A full description of the statistical parameters including central tendency (e.g. means) or other basic estimates (e.g. regression coefficient) AND variation (e.g. standard deviation) or associated estimates of uncertainty (e.g. confidence intervals)
☐	☒	For null hypothesis testing, the test statistic (e.g. *F*, *t*, *r*) with confidence intervals, effect sizes, degrees of freedom and *P* value noted<br>*Give P values as exact values whenever suitable.*
☒	☐	For Bayesian analysis, information on the choice of priors and Markov chain Monte Carlo settings
☒	☐	For hierarchical and complex designs, identification of the appropriate level for tests and full reporting of outcomes
☒	☐	Estimates of effect sizes (e.g. Cohen's *d*, Pearson's *r*), indicating how they were calculated

Our web collection on statistics for biologists contains articles on many of the points above.

Software and code

Policy information about availability of computer code

Data collection	Bruker’s DIFFRAC.Measurement Center V7.5.0 was used to collect X-ray diffraction spectra, nEXt version 1.9 was used for collection of X-ray fluorescence data, TopSpin version 3.6.5 was used to collect 27Al nuclear magnetic resonance data, Cary WinUV Simple Reads version 5.1.3.1042 was used for ultraviolet-visible spectroscopy, Thermo’s XCalibur version 4.1 was used for liquid chromatography mass spectrometry, SSRL’s MicroEXAFS Data Collector 2.0 was used for X-ray absorption near edge structure spectroscopy data.
Data analysis	Peak detection and identification for X-ray diffraction spectra was performed on Bruker’s DIFFRAC.EVA V6.0.0.7. Spectra and peak detection for nuclear magnetic resonance data was performed on MestreNova version 15.0.1. Linear combination fitting for X-ray absorption near edge structure spectra was performed on the ATHENA module of Demeter version 0.9.26. Data analysis for µ-X-ray fluorescence imaging was performed on SMAK version 2.03. Michaelis-Menten model fitting was performed using GraphPad Prism 9.4.0. Maps were created using QGIS version 3.24.

For manuscripts utilizing custom algorithms or software that are central to the research but not yet described in published literature, software must be made available to editors and reviewers. We strongly encourage code deposition in a community repository (e.g. GitHub). See the Nature Portfolio guidelines for submitting code & software for further information.

Data

Policy information about availability of data

All manuscripts must include a data availability statement. This statement should provide the following information, where applicable:

- Accession codes, unique identifiers, or web links for publicly available datasets
- A description of any restrictions on data availability
- For clinical datasets or third party data, please ensure that the statement adheres to our policy

The authors declare that the data supporting the findings of this study are available within the manuscript, the supplementary information files, and as Datasets uploaded to Oak Ridge National Lab Distributed Active Archive Center for Biogeochemical Dynamics (ORNL DAAC) depository under doi: 10.13139/ORNLNCCS/2221769.

Research involving human participants, their data, or biological material

Policy information about studies with human participants or human data. See also policy information about sex, gender (identity/presentation), and sexual orientation and race, ethnicity and racism.

Reporting on sex and gender	n/a
Reporting on race, ethnicity, or other socially relevant groupings	n/a
Population characteristics	n/a
Recruitment	n/a
Ethics oversight	n/a

Note that full information on the approval of the study protocol must also be provided in the manuscript.

Field-specific reporting

Please select the one below that is the best fit for your research. If you are not sure, read the appropriate sections before making your selection.

☐ Life sciences ☐ Behavioural & social sciences ☒ Ecological, evolutionary & environmental sciences

For a reference copy of the document with all sections, see nature.com/documents/nr-reporting-summary-flat.pdf

Ecological, evolutionary & environmental sciences study design

All studies must disclose on these points even when the disclosure is negative.

Study description

This goal of this study was to re-evaluate the role of iron oxide minerals within the phosphorus cycle by measuring the extent of hydrolysis of organic phosphorus compounds reacted with minerals and natural samples taken from a lake and a forest system. Common sediment and soil minerals identified in the natural samples were also reacted with the organic phosphorus compounds to confirm that hydrolysis reactivity of the natural samples was due to iron oxides and not other mineral phases. Seven-day experiments were performed in triplicate with controls in the absence of light. Reaction solutions were analyzed by UV-visible light spectroscopy, high resolution liquid chromatography mass spectrometry, and X-ray absorption near edge structure spectroscopy.

This was a single factor study, where we investigated the effects of different mineral and mineral mixture types on the transformation of organic phosphorus. We investigated seven different minerals (ferrihydrite, goethite, hematite, quartz, mica, kaolinite, and illite), six different mineral mixtures (kaolinite:goethite 1:1, kaolinite:goethite 4:1, quartz:goethite 1:1, quartz:goethite 4:1, ferrihydrite:goethite 1:1, and ferrihydrite:goethite 4:1), and two different environmental samples (a lake sediment and a forest soil). Each of these acted as a different treatment factor. The effects of these different minerals, mineral mixtures, or natural samples on organic phosphorus transformation was also tested across four different organic phosphorus compounds (adenosine triphosphate, adenosine monophosphate, glucose-6-phosphate, and phytate). In total, there were 120 number of experimental units, and 3 complete replicates for each treatment factor.

Research sample

Research samples were solutions of natural samples or minerals reacted with organic phosphorous compounds for seven days. The minerals used in experiments were those determined in the natural samples by X-ray diffraction and iron K-edge X-ray absorption near edge structure spectroscopy: ferrihydrite, goethite, hematite, quartz, mica, kaolinite, illite. These minerals and natural samples were chosen to represent populations of iron-rich soils and sediments. The organic phosphorus compounds used in reactions were chosen due to their common occurrence in sediment or soil systems and within biological systems: adenosine triphosphate, adenosine monophosphate, glucose-6-phosphate, and phytate.

Sampling strategy

No sample size calculation was performed. At least triplicate sampling was conducted because this sample size has been proven to be

Sampling strategy	statistically relevant in laboratory studies. Some treatment factors (i.e. the controls or blanks) utilized more than triplicate sampling due to multiple sets of controls needed for direct comparison between experiments performed at different times.
Data collection	Reactions were stopped by filtration through a 0.2-µm filter or were centrifuged at 4000 rpm for 20 min prior to filtration. An aliquot of the filtrate was analyzed for inorganic phosphate in solution by UV-visible spectroscopy and the remaining filtered sample was frozen at -20 °C until further analysis by high-resolution LC-MS to determine other phosphorus species in solution.<br>Jade Jin Basinski, Dr. Annaleise R. Klein, and Dr. Wiriya Thongsomboon carried out the laboratory experiments, performed UV-visible light spectroscopy to determine inorganic phosphorus in solution, and performed liquid chromatography mass spectrometry analysis to quantify the organic phosphorus compounds in solution. Jade Jin Basinski, Dr. Annaleise R. Klein, Dr. Sharon E. Bone, and Dr. Valerie Mitchell performed X-ray absorption near edge structure spectroscopy for determine the fraction of inorganic phosphorus and organic phosphorus adsorbed onto iron oxide surfaces and natural samples. John T. Shukle, Dr. Greg Druschel, and Dr. Aaron Thompson provided soil and sediment samples and sample characterization data.<br>Reactions were stopped by filtration through a 0.2-µm filter or were centrifuged at 4000 rpm for 20 min prior to filtration. An aliquot of the filtrate was analyzed for inorganic phosphate in solution by UV-visible spectroscopy and the remaining filtered sample was frozen at -20 °C until further analysis by high-resolution LC-MS to determine other phosphorus species in solution.<br>Jade Jin Basinski, Dr. Annaleise R. Klein, and Dr. Wiriya Thongsomboon carried out the laboratory experiments, performed UV-visible light spectroscopy to determine inorganic phosphorus in solution, and performed liquid chromatography mass spectrometry analysis to quantify the organic phosphorus compounds in solution. Jade Jin Basinski, Dr. Annaleise R. Klein, Dr. Sharon E. Bone, and Dr. Valerie Mitchell performed X-ray absorption near edge structure spectroscopy for determine the fraction of inorganic phosphorus and organic phosphorus adsorbed onto iron oxide surfaces and natural samples. John T. Shukle, Dr. Greg Druschel, and Dr. Aaron Thompson provided soil and sediment samples and sample characterization data.
Timing and spatial scale	Experiments were conducted from November 2020 to January 2024. These experiments were performed in a temperature-controlled (293K) and humidity-controlled laboratory (40% RH) for a reaction time of 7 days with controls so there were no differences in the frequency of periodicity of sampling throughout the study.<br>Solution-based data were derived from aliquots of the experimental supernatant (~1 - 10 mL from the total reaction volume of 40 mL).<br>Solid-state data were derived from measurements of a ~1 g sample for X-ray diffraction, X-ray fluorescence, and nuclear magnetic resonance spectroscopy, or from a ~5 mg sample for X-ray absorption near edge structure spectroscopy and µ-X-ray fluorescence imaging.
Data exclusions	No data was excluded from the analyses.
Reproducibility	Experimental findings were verified for reproducibility by the three researchers who performed the experiments or when the standard deviation within the triplicate set was large. Specifically, we verified the repeated sets included reactions of iron oxides (goethite, ferrihydrite, and hematite) with adenosine triphosphate and reaction of minerals (goethite, ferrihydrite, hematite, quartz, mica, kaolinite, illite and controls) with phytate. In both cases, the experimental data were found to be reproducible.
Randomization	Randomization was not relevant to this laboratory study as the study focused on the reactivity of minerals. All samples were sacrificial and were thus independent of each other.
Blinding	This study only involved non-biological research subjects (minerals), thus blinding was not relevant. Data were taken directly from spectroscopic measurements thus there is little room for bias to affect the outcome data. Data analysis consisted primarily of calculating mean +/- standard deviation and model fitting, both of which were automated processes and had minimal opportunity for input of researcher bias.

Did the study involve field work? ☒ Yes ☐ No

Field work, collection and transport

Field conditions	Sediment samples were taken from Missisquoi Bay off of Lake Champlain. The mean annual temperature of the site was 297K. The location for sediment coring is only available by boat and subsequent SCUBA dive.<br>Soil samples were taken from the Calhoun Critical Zone Observatory in the Sumter National Forest. This forest is found in a temperate semi-tropical region with a mean annual precipitation of 1212mm and mean annual temperature of 290K.<br>At both locations, field work included excavation of sediment samples and soil samples for subsequent laboratory analysis and experiments.
Location	Sediment samples were taken from Missisquoi Bay off of Lake Champlain in Vermont (N44°59’33” W73°8’20”). Samples were taken during a SCUBA dive (following AAUS guidelines) with a 0.5m corer.<br>Soil samples were taken from the Calhoun Critical Zone Observatory in the Sumter National Forest in South Carolina (N34°36’33.012” W81°43’40.62”). Samples were taken by backhoe at the 58-86 cm depth.
Access & import/export	Sediment samples were taken from a research designated site during a SCUBA dive following AAUS guidelines by a SCUBA certified researcher, Dr. Gregory K. Druschel. Soil samples were collected at a research site designated for excavation purposes. Both sites do not require permits for sample collection.
Disturbance	All samples were sourced from research sites that are designated for excavation for research activities. There were minimal disturbances to adjacent non-research sites.

Reporting for specific materials, systems and methods

We require information from authors about some types of materials, experimental systems and methods used in many studies. Here, indicate whether each material, system or method listed is relevant to your study. If you are not sure if a list item applies to your research, read the appropriate section before selecting a response.

Materials & experimental systems

n/a	Involved in the study
☒	☐ Antibodies
☒	☐ Eukaryotic cell lines
☒	☐ Palaeontology and archaeology
☒	☐ Animals and other organisms
☒	☐ Clinical data
☒	☐ Dual use research of concern
☒	☐ Plants

Methods

n/a	Involved in the study
☒	☐ ChIP-seq
☒	☐ Flow cytometry
☒	☐ MRI-based neuroimaging
